# Cartilage formation in the pelvic skeleton during the embryonic and early-fetal period

**DOI:** 10.1371/journal.pone.0173852

**Published:** 2017-04-06

**Authors:** Misaki Okumura, Aoi Ishikawa, Tomoki Aoyama, Shigehito Yamada, Chigako Uwabe, Hirohiko Imai, Tetsuya Matsuda, Akio Yoneyama, Tohoru Takeda, Tetsuya Takakuwa

**Affiliations:** 1 Human Health Science, Graduate School of Medicine, Kyoto University, Kyoto, Japan; 2 Congenital Anomaly Research Center, Graduate School of Medicine, Kyoto University, Kyoto, Japan; 3 Department of Systems Science, Graduate School of Informatics, Kyoto University, Kyoto, Japan; 4 Allied Health Science, Kitasato University, Kanagawa, Japan; University of Sheffield, UNITED KINGDOM

## Abstract

The pelvic skeleton is formed via endochondral ossification. However, it is not known how the normal cartilage is formed before ossification occurs. Furthermore, the overall timeline of cartilage formation and the morphology of the cartilage in the pelvis are unclear. In this study, cartilage formation in the pelvic skeletons of 25 human fetuses (crown-rump length [CRL] = 11.9–75.0 mm) was observed using phase-contrast computed tomography and 7T magnetic resonance imaging. The chondrification center of the ilium, ischium, and pubis first appeared simultaneously at Carnegie stage (CS) 18, was located around the acetabulum, and grew radially in the later stage. The iliac crest formed at CS20 while the iliac body’s central part remained chondrified. The iliac body formed a discoid at CS22. The growth rate was greater in the ilium than in the sacrum-coccyx, pubis, and ischium. Connection and articulation formed in a limited period, while the sacroiliac joint formed at CS21. The articulation of the pubic symphysis, connection of the articular column in the sacrum, and Y-shape connection of the three parts of the hip bones to the acetabulum were observed at CS23; the connection of the ischium and pubic ramus was observed at the early-fetal stage. Furthermore, the degree of connection at the center of the sacrum varied among samples. Most of the pelvimetry data showed a high correlation with CRL. The transverse and antero-posterior lengths of the pelvic inlet of the lesser pelvis varied among samples (R^2^ = 0.11). The subpubic angle also varied (65–90°) and was not correlated with CRL (R^2^ = 0.22). Moreover, cartilaginous structure formation appeared to influence bone structure. This study provides valuable information regarding the morphogenesis of the pelvic structure.

## Introduction

Pelvic skeleton formation is an interesting topic in various fields, including anatomy, gynecology, sports medicine, manipulative therapy, occupational ergonomics, biomechanics, anthropometrics, and forensic science [1]. The pelvic skeleton consists of two different functional and structural components: a pair of hip bones (formed forward and to the sides) and the sacrum-coccyx (in the area of the back) [2]. During embryonic and fetal development, the hip bones are described mainly as a proximal component of the hip joint [3–8].

Dislocation and malformation of the hip joint are two of the common congenital anomalies in the skeletal system [4,8]. Thus, from a clinical perspective, hip joint formation has long been a topic of interest. However, hip bone development has not been not as amply described as the femoral bone and joint itself [9]. Furthermore, while the development and morphogenesis of the vertebral column in humans are well documented, the literature regarding descriptions of the sacrum and coccyx is limited [10–13]. Previous studies found that making accurate observations around the sacrum-coccyx was difficult because of the flexure of the samples [12].

Morphometric analysis (pelvimetry) of the pelvic skeleton is performed in various fields [1]. For instance, pelvimetry concerning the pelvic inlet is essential in gynecology clinics because the morphological characteristics are vital for assessing pregnant women. Moreover, pelvimetry is also used in forensic medicine and anthropology, mainly for sex identification [14–19]. Such morphometry is typically relevant for adults and adolescents [16,19]; only limited studies in the fetus exist [14,15].

The timeline of hip bone, sacrum, and coccyx ossification is known, and most of the studies focused on the phases after ossification, for which X-ray images are available [20–22]. However, the morphology and morphometry of the cartilage in the pelvic skeleton are unclear. Pelvic skeletons are formed via mesenchymal condensation and endochondral ossification, and identifying how the normal cartilage is formed before ossification is essential, mainly because cartilaginous structure formation could influence bone structure. In this study, cartilage formation in the pelvic skeleton during embryonic and early-fetal periods was observed.

## Materials and methods

### Human fetal specimens

Approximately 44,000 human fetuses comprising the Kyoto Collection are stored at the Congenital Anomaly Research Center of Kyoto University [23–25]. In most cases, pregnancy was terminated during the first trimester for socioeconomic reasons under the Maternity Protection Law of Japan. Parents provided their verbal informed consent to have the specimens deposited in the collection, and participant consent was documented in each medical record. Written consent was not obtained from all parents. The samples were collected from 1963 to 1995 according to the regulations relevant at each time. The present study, including the consent procedure, was approved by The Committee of Medical Ethics of Kyoto University Graduate School of Medicine, Kyoto, Japan (E986, R0316). The samples were anonymized and de-identified. Some of the specimens (~20%) were undamaged, well-preserved fetuses. The aborted fetuses brought to the laboratory were measured, examined, and staged using the criteria of O’Rahilly and Müller [26]. A total of 29 human fetuses with no obvious damage or anomalies were selected (embryos between Carnegie stage [CS] 17 and CS23, CS17, n = 5; CS18, n = 1; CS19, n = 2; CS20, n = 3; CS21, n = 2; CS22, n = 2; CS23, n = 2; and early fetus (EF) in trimester 1, n = 12). Samples ranged from 6–11 weeks old (approximately 42–77 days) post-fertilization.

EF was defined as a fetus that was more developed than those at CS23 (i.e., with a crown-rump length [CRL] of 34.0–75 mm and age of approximately 8–11 weeks post-fertilization) [27].

### Image acquisition and data analysis

This study employed 3D phase-contrast X-ray tomography (PCXT) image acquisition, as described previously [28]. Briefly, specimens were visualized with a phase-contrast imaging system fitted with a crystal X-ray interferometer [29]. The system was set up at the vertical wiggler beamline (PF BL14C) of the Photon Factory in Tsukuba, Japan. The white synchrotron radiation emitted from the wiggler was monochromated by a double-crystal monochromator using Si(220), expanded horizontally by an asymmetric crystal, and inputted into the imaging system. The generated interference patterns were detected by a large-area X-ray imager composed of a 30-μm scintillator, relay lens system, and water-cooled charge-coupled device camera (36 × 36 mm field of view, 2048 × 2048 pixels per 18 × 18 μm) [30]. The X-ray energy was tuned at 17.8 keV, and an exposure time of 3 s was used to obtain one interference pattern. The average intensity was approximately 300 counts pixel^−1^s^−1^, which allowed for fine observations within a reasonable measurement time.

Magnetic resonance (MR) images were acquired using a 7T magnetic resonance imaging (MRI) system (BioSpec 70/20 USR; BrukerBioSpin MRI GmbH; Ettlingen, Germany) with a 35-mm-diameter quadrature transmit-receive ^1^H volume coil (T9988; Bruker BioSpin MRI GmbH; Ettlingen, Germany). 3D T1-weighted images were acquired using a fast, low-angle shot pulse sequence with the following parameters: repetition time, 30 ms; echo time, 4.037–6.177 ms; flip angle, 40°; field of view, 22.5 × 15.0 × 15.0–42.0 × 28.0 × 28.0 μm^3^; matrix size, 636 × 424 × 424–768 × 512 × 512; and spatial resolution, 35.4–54.7 μm^3^.

Moreover, 3D PCXT was used to acquire images of the samples between CS17 and CS21, and MRI was used for the samples at CS22 and later. The image acquisition method was selected based on sample resolution and size. Thus, 3D PCXT was used to acquire images at a higher resolution. CS21 embryos represented the upper limit in terms of size (approximately 20 x 20 x 20 mm). For samples with larger sizes, images were obtained using MRI. 3D PCXT and MRI data from the samples were analyzed using reconstructed 3D and serial 2D images, respectively. The pelvic structure images in all samples were reconstructed using Amira software version 5.4.5 (Visage Imaging GmbH; Berlin, Germany).

### Categorization of the connection and articulation of the cartilage

**Fig 1:**
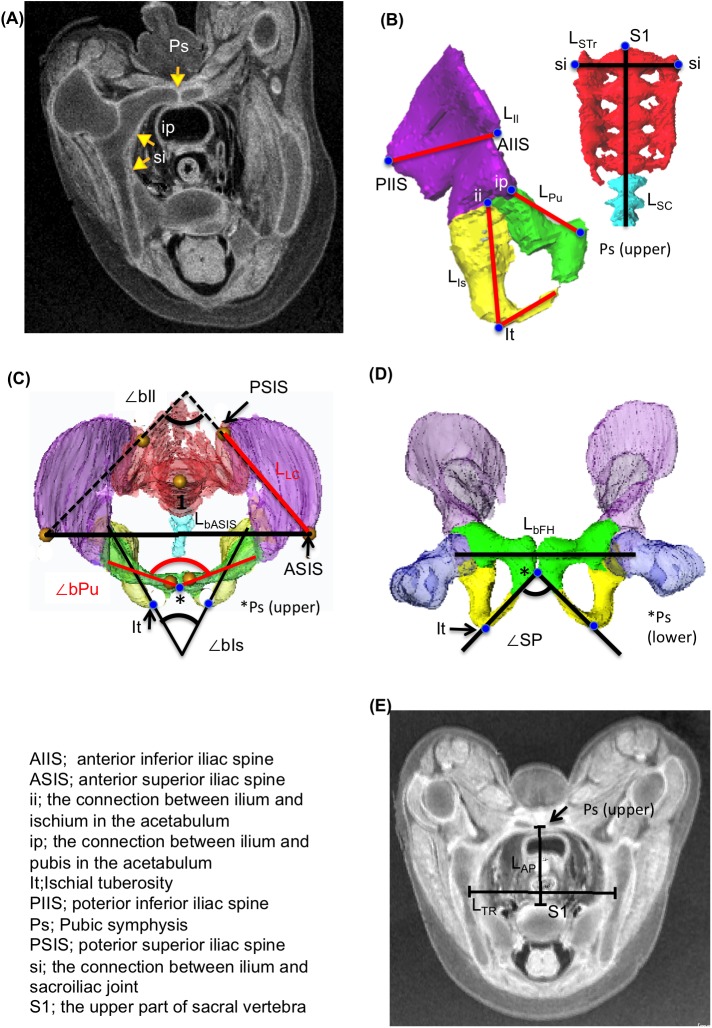
Measurements of Each Component of the Hip Bone, Sacrum, and Coccyx, and Pelvimetry of the Greater and Lesser Pelvis. (**A**) Representative transverse section with MR image showing the connections and articulations in the pelvic skeleton. Pubic symphysis (Ps) was categorized as grade 1, the connection between ilium and pubis in the acetabulum (ip) as grade 3, and the sacroiliac joint (si) as grade 1. (**B**) Growth of the components of the hip bone, sacrum, and coccyx. The longitudinal length of the pubis, ischium, ilium, and sacrum and coccyx was indicated as L_Pu_, L_Is_, L_Il_, and L_SC,_ respectively. Transverse length of the sacrum was indicated as L_STr_. (**C, D**) Pelvimetry for the greater pelvis: The length between bilateral anterior superior iliac spine (L_bASIS_), length between bilateral femoral heads (L_bFH_), length of the lateral conjugate (L_LC_) or the length between ASIS and posterior superior iliac spine (PSIS), subpubic angle (∠SP), and angle of bilateral superior pubic ramus (∠bPu), bilateral ischium (∠bIs), and bilateral ilium body (∠bIl) as shown in the cranial (**C**) and frontal (**D**) views of the 3D reconstructed pelvic skeleton. (**E**) Pelvimetry for the lesser pelvis: the transverse (L_TR_) and antero-posterior (L_AP_) diameters of the pelvic inlet as shown in the MR image (transverse section).

MRI was used to assess the following: the connection of the pubis, ilium, and ischium to the acetabulum and ramus; the connection of each vertebra to the articular column of the sacrum and coccyx; the articulation of the pubic symphysis; and the acetabular sacroiliac joints (Fig 1A). The degree of angulation was categorized as follows: both cartilages of the hip bones, sacrum, and coccyx were totally separated (0), contiguous from the surface (1), partially united (2), and united (3).

### Morphometric analysis

For quantitative evaluation, each component of the hip bones was measured and pelvimetry of the greater pelvis and lesser pelvis was performed using Amira software (Table 1). To evaluate the growth of the pubis, ischium, ilium, sacrum, and coccyx, their longitudinal length (L_Pu_, L_IS_, L_Il_, and L_SC_, respectively) and the transverse maximum length of the sacrum (L_STr_) were measured (Fig 1B). The angle of bilateral superior pubic ramus (∠bPu), bilateral ischium (∠bIs), and bilateral ilium body (∠bIl) was also measured (Fig 1C). For pelvimetry of the greater pelvis, the length between bilateral anterior superior iliac spines (ASIS) (L_bASIS_), length between bilateral center of femoral heads (L_bFH_), length of the lateral conjugate (L_LC_; defined as the length between ASIS and posterior superior iliac spine (PSIS),) and subpubic angle (∠SP) were measured (Fig 1C and 1D). For pelvimetry of the lesser pelvis, the transverse maximum diameter (L_TR_) and antero-posterior diameter (L_AP_) of the pelvic inlet, defined as the plane that included the upper part of sacral vertebra (S1) and bilateral upper part of the pubic symphysis, were measured (Fig 1E). The correlation between each pelvimetry and CRL was analyzed.

**Table 1:** Summary of fetal pelvis morphometry.

	Symbol	Description	Figure number	
			Definition	Results
Growth of the components of the hip bone				
	Lpu	Longitudinal length of the pubis [between upper Ps and ip]	1B	5A
	Lis	Longitudinal length of the ischium [between ii and It plus inferior ramus]	1B	5A
	Lil	Longitudinal length of the ilium [between PIIS and AIIS]	1B	5A
	LSC	Longitudinal length of the sacrum and coccyx [between sv and tip of coccyx]	1B	5A
	LSTr	Transverse maximum length of the sacrum [between bilateral si]	1B	5A
	∠bPu	Angle of the bilateral superior pubic ramus [between bilateral ip-upper Ps]	1C	5B
	∠bIs	Angle of the bilateral ischium [between bilateral segments ii-It]	1C	5B
	∠bIl	Angle of the bilateral ilium body	1C	5B
Pelvimetry of the greater pelvis				
	LbASIS	Length between the bilateral ASIS	1C	7A
	LbFH	Length between the bilateral center of femoral heads	1D	7A
	LLC	Length between the ASIS and PSIS (lateral conjugate)	1C	7B
	∠SP	Subpubic angle [between bilateral segment It-lower Ps]	1D	7C
Pelvimetry of the lesser pelvis				
	LTR	Transverse maximum diameters of the pelvic inlet	1E	8A
	LAP	Antero-posterior diameters of the pelvic inlet [between upper Ps and sv]	1E	8A

AIIS; anterior inferior iliac spine, ASIS; anterior superior iliac spine, ii; the connection between the ilium and ischium in the acetabulum, ip; the connection between the ilium and pubis in the acetabulum, It; ischial tuberosity, PIIS; poterior inferior iliac spine, Ps; pubic symphysis, PSIS; posterior superior iliac spine, si; the connection between the ilium and sacroiliac joint.

### Correlation analyses

The correlation between each morphometric parameter and the CRL was analyzed with the least squares method using Excel 2011 for Macintosh (Microsoft Corp., WA, USA).

## Results

### Cartilage formation in the pelvic skeleton

Cartilage formation in each part of the pelvic skeleton was analyzed using the reconstructed images obtained from 3D PCXT and MRI.

#### Hip bone

**Fig 2:**
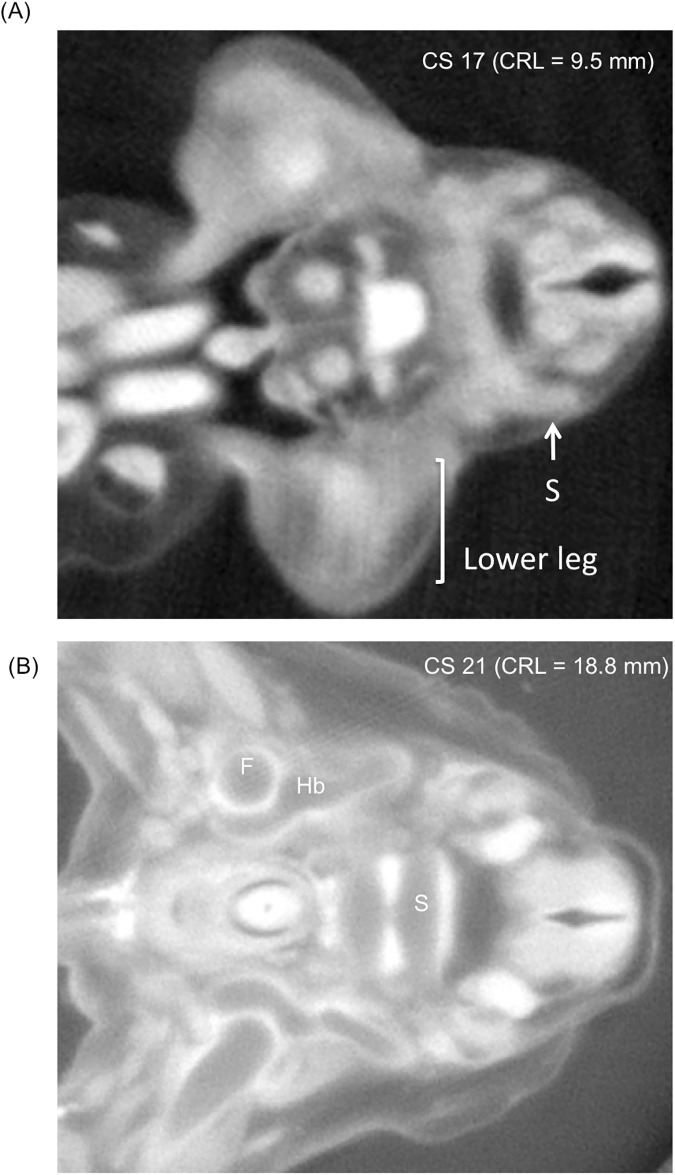
**Representative Transverse Section with PCXT image at CS17 (A) and CS 21 (B). A)** A vague mass with relatively high density was detected around the proximal part of the lower leg, which may correspond to mesenchymal condensations that form into a template for later chondrification. B) Chondrification of the hip bones, femur, and sacrum was detected.

**Fig 3:**
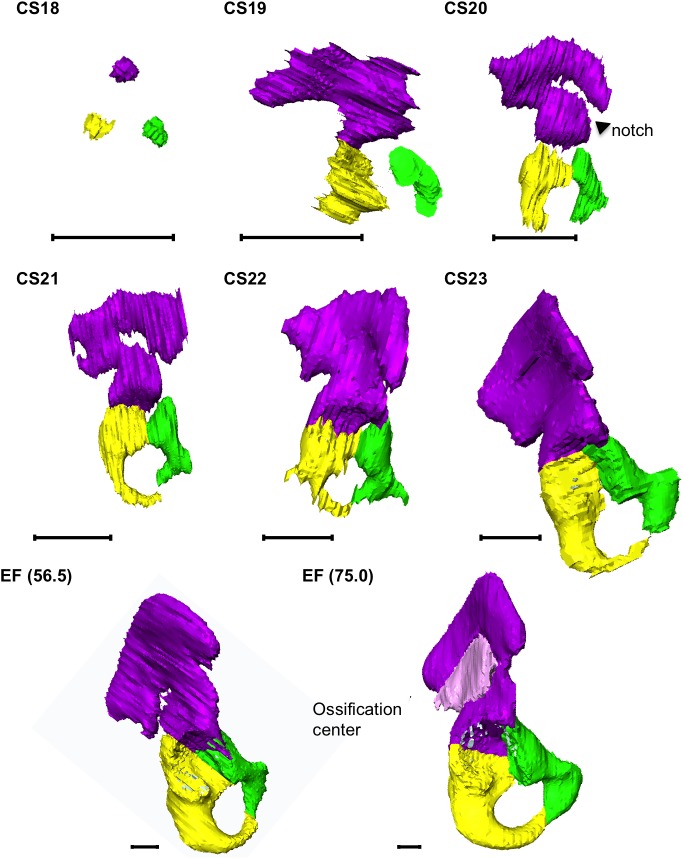
3D Reconstruction of Cartilage Formation in the Hip Bone. The acetabulum was located at the center of the illustration. CS, Carnegie stage; EF, early fetus. Green, pubis; Purple, ilium; Yellow, ischium. Pink in EF (75 mm) indicates the ossification center in the ilium. The number in parentheses indicates the CRL (mm). The scale bar indicates 1 mm.

None of the three components of the hip bone (i.e., pubis, ischium, and ilium) was chondrified at CS 17, while vague mass with relatively high density was detected around the proximal part of the lower leg. The mass may correspond to mesenchymal condensations, which form into a template for later chondrification. Chondrification was detected only in the sacrum and coccyx at CS17 (Fig 2A). The chondrification center of the three components first appeared simultaneously at CS18 and was located around the acetabulum (Fig 3). Chondrification of the hip bone became more apparent after CS19 (Fig 2B).

The ilium grew toward the sacrum at CS19 (Fig 3), while the iliac crest formed at CS20. Consequently, the notch formed transitionally at CS20 and CS21. The iliac body formed a discoid at CS22. The ossification center was detected in the EF, with a 49.5-mm CRL. The ischium grew medial-caudally, forming the ischemic body, and, subsequently, medial-cranially at CS22, with ischial tuberosity formation. The pubis grew medially, forming the ramus superior. The pubic body formed at CS21 and the ramus inferior at CS22. The ischial spine and ramus inferior became closer. Eventually, unification and formation of the obturator foramen was first detected in an early fetus with a 34.0-mm CRL.

#### Sacrum and coccyx

**Fig 4:**
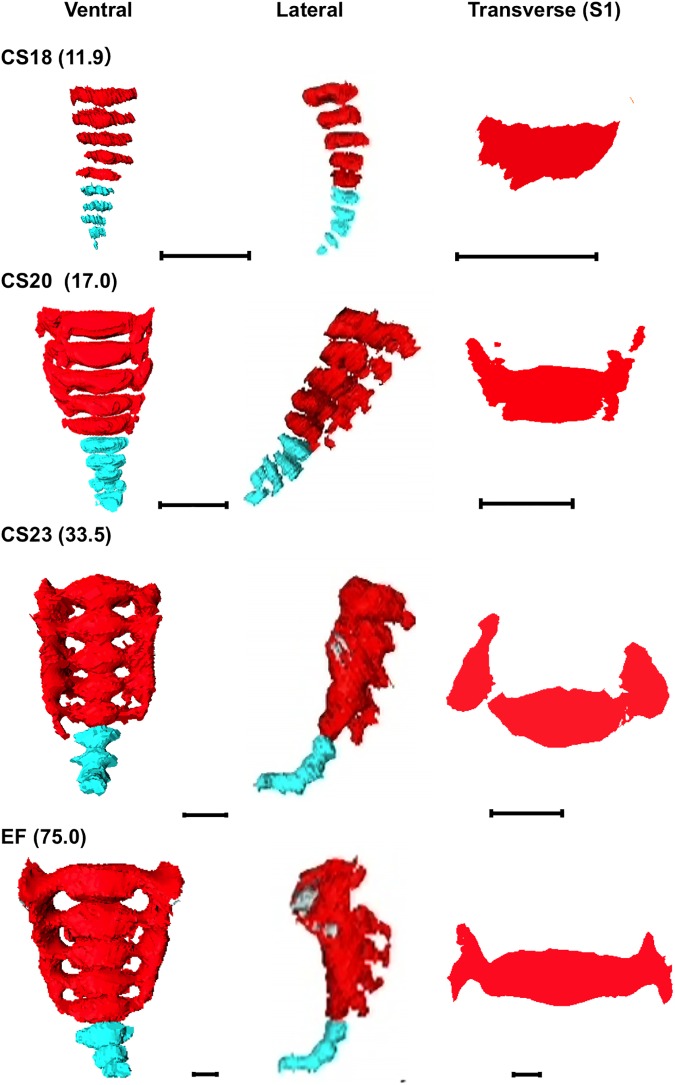
Cartilage Formation in the Sacrum and Coccyx. Three-D reconstruction of the sacrum and coccyx. Ventral view, lateral view, and transverse section at S1 are shown. CS, Carnegie stage; EF, early fetus. Light blue, coccyx; Red, sacrum. Scale bar indicates 1 mm.

Five sacral and five coccyx vertebrae were separated, similar to the vertebral column in the upper thoracic and lumbar regions, at CS18 (Fig 4). During development, the columns became broad and the vertebra and ala of the sacrum fused. The ala formed the articular column until CS23, which articulates with the ilium forming the sacroiliac joint (Table 2). The degree of connection at the center of the sacrum varied between samples. Eight of 12 EF samples showed that the vertebral column structure remained in the sacrum. The articulation between the sacrum and coccyx was found in most of the samples, and both remained fused in two EF samples. Four coccyx vertebrae were detected in only two samples.

**Table 2:** Connection of the Sacrum-Coccyx Column in Each Sample.

Sample ID		1	2	3	4	5	6	7	8	9	10	11	12	13	14	15	16	17	18	19	20	21	22	23	24
CRL (mm)		11.9	14.9	15.5	17.0	17.0	17.0	18.8	19.7	22.0	24.5	26.8	33.5	34.0	37.2	40.0	43.5	49.5	51.0	56.5	56.5	62.0	66.0	69.0	75.0
Carnegie stage		18	19	20	19	20	20	21	21	22	22	23	23	EF	EF	EF	EF	EF	EF	EF	EF	EF	EF	EF	EF
vertebra	S1-2	1	1	1	1	1	1	1	1	1	1	3	3	1	3	3	1	3	1	1	3	3	1	1	3
	S2-3	1	1	1	1	1	1	1	1	1	1	1	3	1	3	3	1	3	3	3	3	3	1	1	3
	S3-4	1	1	1	1	1	1	1	1	1	1	1	3	1	3	3	1	3	3	3	3	3	3	1	3
	S4-5	1	1	1	1	1	1	1	1	1	1	1	3	1	3	1	1	3	3	3	3	3	3	1	3
	S5-C1	1	1	1	1	1	1	1	1	1	1	1	1	1	1	1	1	1	1	1	3	1	1	1	3
	C1-2	1	1	1	1	1	1	1	1	1	1	1	3	1	3	1	1	3	3	3	3	3	3	1	3
	C2-3	1	1	1	1	1	1	1	1	1	1	1	3	1	3	1	3	3	3	3	3	3	3	3	3
	C3-4	1	1	1	1	1	1	1	1	1	1	1	3	1	3	1	3	3	3	3	3	3	3	3	3
	C4-5	1	1	1	1	1	1	1	ND	1	1	1	3	1	3	3	3	3	3	3	ND	3	3	3	3
articular	S1-2	1	1	1	1	1	1	2/1	2	0	1/2	3	3	3	3	3	3	3	3	3	3	3	3	3	3
column	S2-3	1	1	1	1	1	1	0	0	3/2	1/2	3	3	3	3	3	3	3	3	3	3	3	3	3	3
(R/L)	S3-4	1	1	1	1	1	1	2	0	3/2	3/2	3	3	3	3	3	3	3	3	3	3	3	3	3	3
	S4-5	1	1	1	1	1	1	2	0	3/0	3/2	3	3	3	3	3	3	3	3	3	0	3	3	3	3

Degree of connection was categorized as follows: both cartilages of the hip bones were totally separated (0), contiguous from the surface (1), partially united (2), and united (3). ND, the fifth coccyx was not detected. When the degree of connection was different between the right and left side, the values are shown as “degree of right side/degree of left side.” C, coccyx; EF, early fetus; S, sacrum.

Neural processes extended dorsally on each side of the neural tube during the fetal period and later became the neural arch. Moreover, the connection of each vertebra and the articular column in each sample is shown in Table 2. Both the center and ala of the vertebrae were connected at CS23. The articular column formed in all samples after CS23. However, the vertebral column connections varied among samples.

### Growth speed and direction of the cartilages

**Fig 5:**
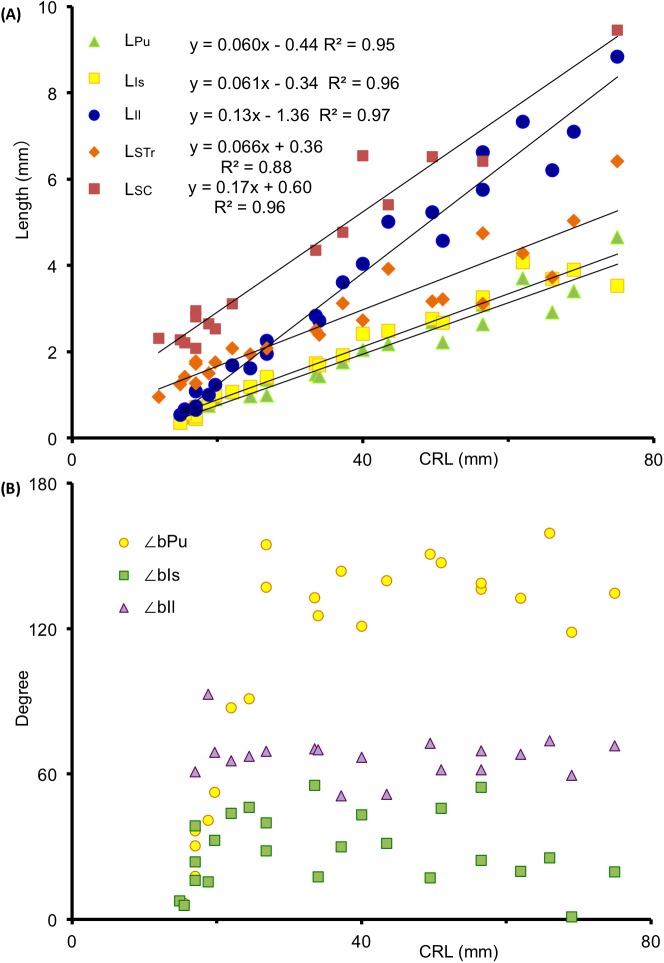
The Growth Speed of Each Component of the Cartilage. (**A**) Longitudinal length of the ilium (L_Il_), ischium (L_Is_), and pubis (L_Pu_), and longitudinal (L_SC_) and transverse (L_STr_) length of the sacrum-coccyx according to CRL. The lengths measured are shown in Fig 1A. (**B**) The angle of the bilateral superior pubic ramus (∠bPu), bilateral ischium (∠bIs), and bilateral ilium (∠bIl) measured according to CRL, indicating the orientation of the growth.

L_Il_, L_Is_, and L_Pu_ correlated with CRL (R^2^ = 0.97, 0.96, and 0.95, respectively; Fig 5A). L_STr_ and L_SC_ correlated with CRL (R^2^ = 0.88 and 0.96, respectively). The growth of the sacrum and coccyx preceded that of the hip bone in the earlier stages, with a growth rate comparable to that of the ischium and pubis. The growth rate was greater in the ilium than that in the sacrum-coccyx, pubis, and ischium. The pubis, ischium, and ilium grew medially during the observation period, although their growth orientation differed by angle, i.e., ∠bPu was around 120–150°, ∠bIs around 10–50°, and ∠bIl around 60–70°(Fig 5B). ∠bPu, ∠bIs, and ∠bIl varied among samples.

### Pelvic ring formation with connection and articulation of the cartilage

**Fig 6:**
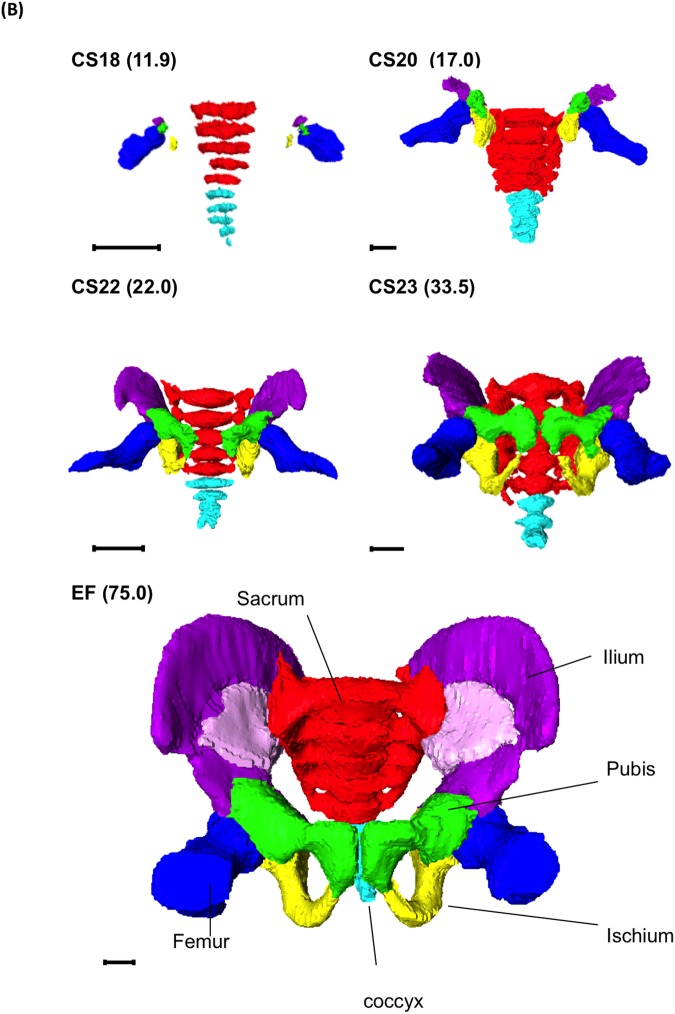
Pelvic Ring Formation. Three-D reconstruction of cartilage formation in the pelvic skeleton (frontal view). Blue, femur; Green, pubis; Light blue, coccyx; Orange, aorta and common iliac arteries. Purple, ilium; Red, sacrum; Yellow, ischium. The number in parentheses indicates the CRL (mm). The scale bar indicates 1 mm. See also S1–S6 Videos.

The articulations and connections of each cartilage were required for pelvic skeleton formation. The detailed timetable of the connection and/or articulation is shown in Table 3. The sacroiliac joint articulated at CS21. The articulation of the pubic symphysis, connection of articular column in the sacrum, and Y-shape connection of the three parts of the hip bones to the acetabulum were observed at CS23; the connection of the ischium and pubic ramus formed in the early-fetal period. Based on the formation of the articulation and connection, the pelvic ring is formed and may contribute to the final stable structure (Fig 6, S1–S6 Videos).

**Table 3:** Connections and Articulations of Each Cartilage in Each Sample.

Sample ID		1	2	3	4	5	6	7	8	9	10	11	12	13	14	15	16	17	18	19	20	21	22	23	24
Crown-Ramp length (mm)		11.9	14.9	15.5	17.0	17.0	17.0	18.8	19.7	22.0	24.5	26.8	33.5	34.0	37.2	40.0	43.5	49.5	51.0	56.5	56.5	62.0	66.0	69.0	75.0
Carnegie stage		18	19	20	19	20	20	21	21	22	22	23	23	EF	EF	EF	EF	EF	EF	EF	EF	EF	EF	EF	EF
ossification center in ilium		-	-	-	-	-	-	-	-	-	-	-	-	-	-	-	-	+	-	-	+	+	+	+	+
Cavity in hip joint (R/L)		-	-	-	-	-	-	-	-	-/*	-	*	-/*	*/+	+	*/+	+	+	+/*	+	+	+	+	+	+
acetabulum	ilium-pubis	0	0	0	0	0	1	0	1	3	1	3	3	3	3	3	3	3	3	3	3	3	3	3	3
(Y-shape)	pubis-ischium	0	0	0	3/0	0	1	3/2	3	3	3	3	3	3	3	3	3	3	3	3	3	3	3	3	3
(R/L)	ischium-ilium	0	0	1/3	0	0	3/1	2	3	1	3	3	3	3	3	3	3	3	3	3	3	3	3	3	3
ischiopubic ramus (R/L)		0	0	0	0	0	0	0	0/3	3/0	0	0	0	3	3	3	3	3	3	0/3	3	3	3	3	3
pubic symphysis		0	0	0	0	0	0	0	0	0	0	0	1	1	1	1	1	1	1	1	1	1	1	1	1
sacroiliac joints (R/L)		0	0	0	0	0	0	0	*	1	1	1	1	1	1	1	1	1	1	1	1	1	1	1	1

Degree of angulation was categorized as follows: both cartilages of the hip bones were totally separated (0), contiguous from the surface (1), partially united (2), and united (3).(-) not formed, (+) detected, (*) hard to determine. When the degree of connection was different between the right and left side, values are shown as “degree of right side/degree of left side.” EF, early fetus.

### Pelvimetry

**Fig 7:**
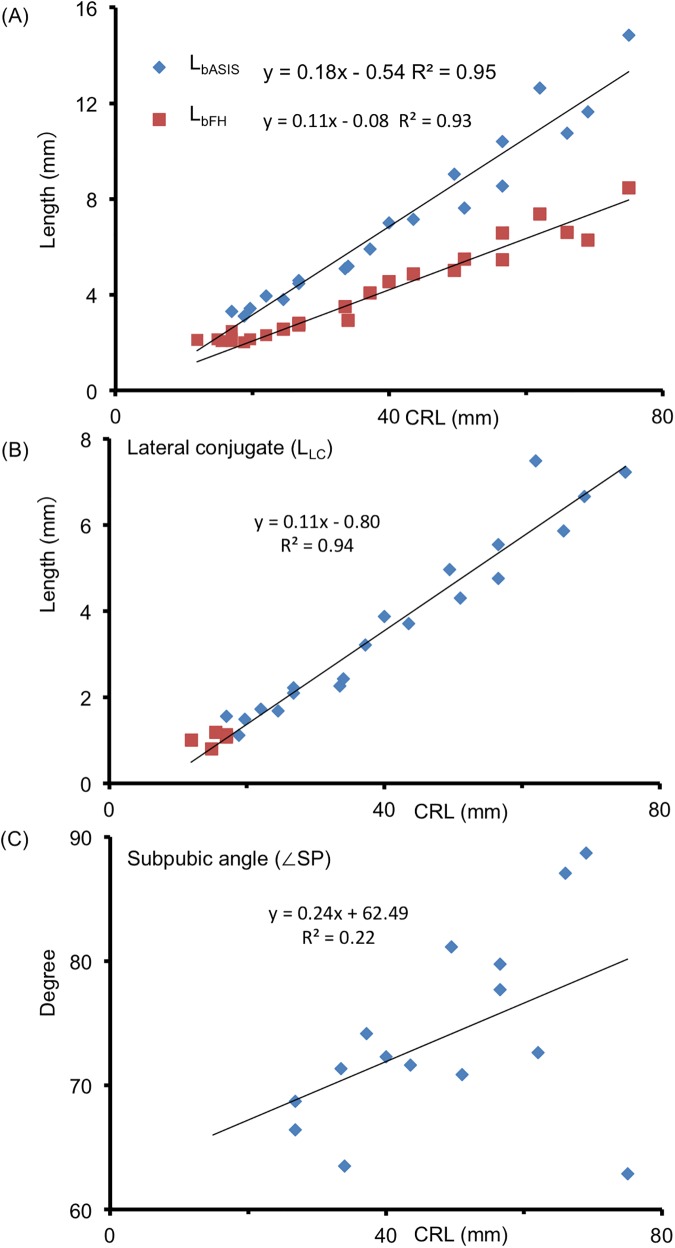
Pelvimetry of Greater Pelvis. (**A**) Length between bilateral anterior superior iliac spine (L_bASIS_) and length between bilateral femoral heads (L_bFH_) according to CRL. (**B**) Length of the lateral conjugate (L_LC_) according to CRL. (**C**) Subpubic angle (∠SP) according to CRL. The lengths and angle measured are shown in Fig 1B and 1C.

For the greater pelvis, L_bASIS_, L_bFH_, and L_LC_ increased linearly according to the CRL, which showed high correlations (R^2^ = 0.95, 0.93, and 0.94, respectively; Fig 7A and 7B). The subpubic angle was approximately 65–90° during the observation period. The angle was not correlated with CRL (R^2^ = 0.22; Fig 7C).

**Fig 8:**
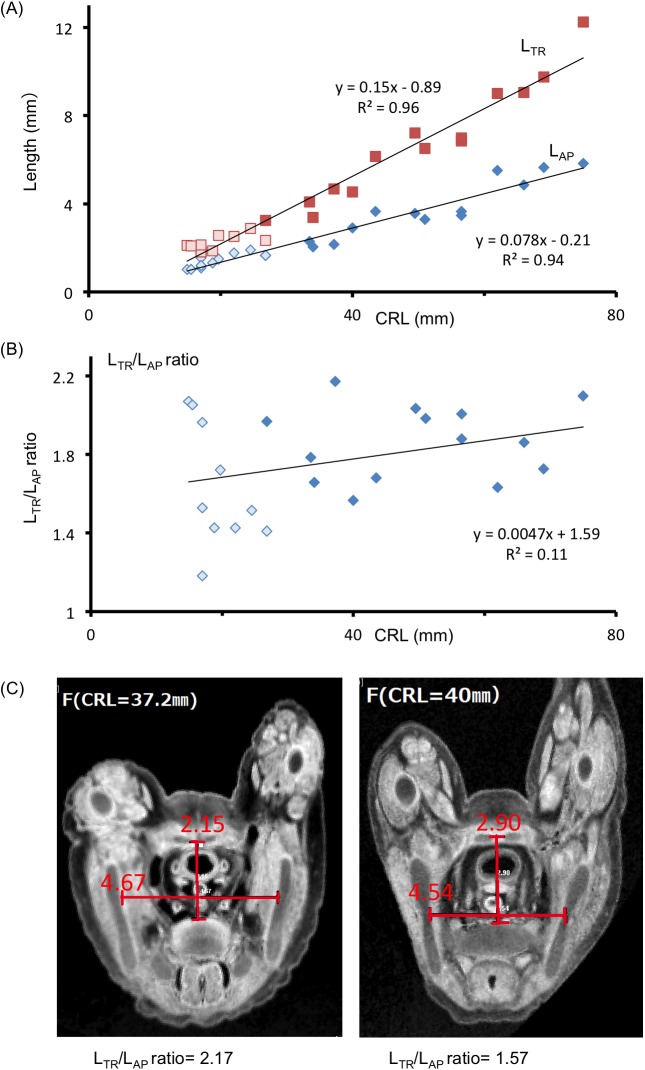
Pelvimetry of the Lesser Pelvis. (**A**) Transverse diameter (L_TR_) and antero-posterior diameter (L_AP_) of the pelvic inlet according to CRL. The lengths measured are shown in Fig 1D. (**B**) L_TR_/L_AP_ ratio according to CRL. (**C**) Two representative cross-sectional MR images of the pelvic inlet of the lesser pelvis. Note the variation of the L_TR_/L_AP_ ratio between the two samples.

For the lesser pelvis, L_TR_ and L_AP_ of the pelvic inlet were measured. Both L_TR_ and L_AP_ increased linearly according to CRL, which showed significant correlations (R^2^ = 0.96 and 0.94, respectively; Fig 8A). However, L_AP/TR_ ratio varied among samples (R^2^ = 0.11; Fig 8B and 8C), which reflects the variable pelvic inlet formation of the lesser pelvis.

## Discussion

Pelvic skeleton formation via mesenchymal condensation and endochondral ossification is well understood. The first ossification center appears in the ilium in the early-fetal period [20,21]. Ossification occurs from multiple sites and continues after birth until adolescence [31]. While most previous studies focused on the phases after ossification for which X-ray images are available, studies concerning structural formation during the cartilaginous stage are limited [10,11,32–34]. This could be attributed to the analysis required, i.e., either through 3D reconstruction from serial histological sections or cross-sectional observation after potassium hydroxide clearance and alizarin red staining instead of simple X-ray images [20,21]. A previous study revealed that the cartilaginous structure differs from the bony structure; however, precise quantitative and morphometric analysis have not yet been performed [10,11,32,33]. Thus, the present study is valuable because the morphogenesis of the pelvic structure in the cartilaginous stage is clearly shown in 3D, and the timeline of the connection and articulation of each component and the results of the morphometric analysis ware presented.

Boucher [14] described differences in the fetal pelvis with respect to sex. The subpubic angle, width, and depth of the sciatic notch differed with sex; however, the growth of the ischium and pubis and the ischium-pubis indices did not differ with age. Moreover, Haque *et al*. [15] noted dimorphism in the subpubic angle (58–64°) of human fetuses at 14–22 weeks, indicating sex differences. In the present study, the subpubic angle varied too. However, sex was only recognized in limited larger samples with CRL > 44 mm; sex can be discriminated by external genitalia at the early-fetal stage [35]. Therefore, variability in subpubic angle may, in part, result from the sex of the samples.

L_AP_ and L_TR_ at the pelvic inlet of the lesser pelvis were highly correlated with CRL, while L_AP/TR_ ratio varied. L_AP_, L_TR_, and L_AP/TR_ ratio directly indicate the size and form of the bony birth canal in women; thus, these parameters have gynecological importance. Furthermore, these morphometric data show sex and individual differences in adults and adolescents. It should be noted that L_AP/TR_ variations were already present in the samples with cartilage formation; however, whether the variations observed in the cartilage structure resulted in variations in bone structure was not determined in this study.

The connections and articulations of the cartilage in the pelvis are essential for pelvic ring formation. In this study, 3D reconstruction was employed to aid in the understanding of morphogenesis. Consequently, we found that the connection and articulation formed to pelvic ring in a limited period between CS22 and CS23, which corresponds to approximately 54–60 days after fertilization. The formation of the pelvic ring structure in this proper period should be necessary for the initiation of effective fetal movement, which may induce mechanical forces and affect normal skeletal development [36]. On the other hand, fetal movements may also explain some of the variation observed between samples [36].

In the sacrum and coccyx, the connections and articulations varied in the cartilaginous stages. This observation could be attributed to the individual differences in the timeline (delay of connection), anomalies (non-union until adults), or both. Furthermore, variation in the number of coccyx vertebrae and the fusion of the sacrum and coccyx were detected in the present study.

Apart from timeline variations, anomalies in the sacrum and coccyx region are occasionally recognized after birth. For instance, connection defects of the neural arch are well known in this region, and are incidentally detected by radiography. Examples include spina bifida occulta, a failure in the completion of the neural arch in the lumbosacral region that is typically found in 95% of 2-year-old children and in approximately 20% of adults [37].

Moreover, the cartilaginous structure could influence bone structure formation, as ossification occurs as if the cartilage structures are blue prints replaced by bone structures [31]. Connection defects in the cartilage structure may affect the bony structure. Further study is warranted to reveal the extent of the effect of the variation of the cartilaginous structure on bony structures.

The basic pattern of chondrification described for the humerus includes five phases of development [34]. Similar phases of cartilage differentiation are also present in the ilium [33]. In the present study, such phases were not discernable with data from PCXT and MRI, though the feature of the signals changed during development, such as intensity, clarity of the boarder, heterogeneity in the cartilage, and so on. Further study concerning the relationship between histological change during chondrification and the features of the signals from PCXT and MRI may help to elucidate these issues.

Mesenchymal cell condensation before chondrification is an important issue because it could affect the morphogenesis of the cartilage. Cell condensations, however, were not clearly detected in PCXT. One reason for this is that PCXT is not usually used to detect soft tissue. Another is that the samples were too small to resolve the region with PCXT. Further improvement of the PCXT acquisition may make it possible to analyze the phases before cartilage formation.

Precise pelvimetry of the cartilaginous structure could lead to prenatal diagnosis in the future. For instance, Lee *et al*. [37] reported that the iliac angle is larger in pregnancies with Down syndrome than in those without the disorder during the second trimester (at an average of 20.6 weeks of gestation). In the present study, the iliac angle in the cartilaginous stage was measured and showed no variation among samples. Thus, whether the iliac angles in samples with Down syndrome have already been different in the cartilaginous stage could be of particular interest.

In conclusion, with the methods used and the data obtained, this study provides valuable information concerning the morphogenesis of the pelvic structure.

## Supporting information

S1 Video The Representative Video of the Pelvic Structure at CS19. (AVI)

S2 Video The Representative Video of the Pelvic Structure at CS20. (AVI)

S3 Video The Representative Video of the Pelvic Structure at CS21. (AVI)

S4 Video The Representative Video of the Pelvic Structure at CS22. (AVI)

S5 Video The Representative Video of the Pelvic Structure at CS23. (AVI)

S6 Video The Representative Video of the Pelvic Structure at the Early-Fetal Period (CRL = 75 mm). (AVI)
